# Pigeon Inspired Optimization with Encryption Based Secure Medical Image Management System

**DOI:** 10.1155/2022/2243827

**Published:** 2022-08-08

**Authors:** B. T. Geetha, Prakash Mohan, A. V. R. Mayuri, T. Jackulin, J. L. Aldo Stalin, Varagantham Anitha

**Affiliations:** ^1^Department of ECE, Saveetha School of Engineering, SIMATS, Saveetha University, Chennai, India; ^2^Department of Computer Science and Engineering, Karpagam College of Engineering, Coimbatore, India; ^3^SCSE, VIT Bhopal University, Bhopal, India; ^4^Department of CSE, Panimalar Engineering College, Chennai, India; ^5^Department of Information Technology, Sona College of Technology, Salem, India; ^6^Department of Computer Science, Institute of Technology, Hawassa University, Hawassa, Ethiopia

## Abstract

Presently, technological advancements in the healthcare sector pose a challenging problem relevant to the security and privacy of health-related applications. Medical images can be considered significant and sensitive data in the medical informatics system. In order to transmit medical images in an open medium, the design of secure encryption algorithms becomes essential. Encryption can be considered one of the effective solutions for accomplishing security. Although numerous models have existed in the literature, they could not adaptable to the rising number of medicinal images in the health sector. At the same time, the optimal key generation process acts as a vital part in defining the performance of the encryption techniques. Therefore, this article presents a Pigeon Inspired Optimization with Encryption-based Secure Medical Image Management (PIOE-SMIM) technique. The proposed PIOE-SMIM approach majorly concentrates on the development of secret share creation (SSC) and the encryption process. At the initial stage, the medical images are converted into a collection of 12 shares using the SSC approach. In addition, an elliptic curve cryptography (ECC) scheme is employed for the encryption process. In order to optimum key creation procedure in the ECC model, the PIO technique is exploited with the aim of maximizing PSNR. Finally, on the receiver side, the decryption and share reconstruction processes are performed to construct the original images. The PIOE-SMIM model displayed an enhanced PSNR of 59.37 dB in image 1. Improved PSNR of 59.53 dB is given for image 5 using the PIOE-SMIM model. For demonstrating an enhanced performance of the PIOE-SMIM method, a widespread experimental study is made and the results highlighted the supremacy of the PIOE-SMIM model over other techniques.

## 1. Introduction

Most healthcare providers in recent times have implemented any procedure of electronic mode of medical record system [1]. For reducing the cost of medical imaging (MI) structures by means of front-end ownership and IT maintenance cost burdens, many health practices have changed to outsource options and save MI data from the centralization databases on a third-party cloud which is not completely trustworthy and could be probably presented at remote areas. Though, if the MI data is saved on a third-party cloud and probably distrusted cloud, security, and secrecy in relation to the safeguarding of data and data access become critical problems [2, 3]. Security and secrecy problems of the healthcare cloud emerge primarily from the inherent property of cloud computing (CC), namely heterogeneity and service composition and consolidation, multitenancy, and dynamic resources [4].

Initially, if the medical activities deploy the cloud memory services to save their MI data on a remote cloud [5], these medical practices activities needed to encounter the actual risk that their MI data are being widely open to the cloud service provider by which unlawful accesses for invading the secrecy and confidentiality of the saved medicinal data [6]. Second, the medicinal data from various medicinal practices are frequently held in a similar memory cloud for data accessing and processing [7]. Confirming the security purpose and secrecy of patients and other private information is an ongoing research area. Hence, we definitely needed an encryption structure that delivers a very effective method for controlling data accessibility on the basis of user attributes (or privileges), relatively more than their identities. The healthcare cloud is a mission-critical cloud service system that needs the strongest security and secrecy guarantees for medicinal practices with reference to both data as well as access [8, 9]. First, for the sake of preventing information leakages from the saved data, all health data saved on the healthcare cloud must be encoded [10]. Pigeons are simple and intelligent birds that can fly long distances in search of food and then return home without becoming disoriented. This move encouraged researchers, who concluded that pigeons navigate by using the Earth's magnetic field, differences in sun altitude, and memory of a few landmarks. The success of any algorithm is guaranteed when the algorithm can explore and exploit the problem domain globally. Probing PIO on multiple applications allows us to gain a deeper understanding of the method. Pigeons are known to travel large distances in quest of food. They can fly through a forest as well as 759 International Journal of Engineering and Technology through wide space. Another intriguing feature is that the pigeons can find their way back to their home by sensing the Earth's magnetic field, the sun's height, and visual cues such as landmarks. Leading pigeons in the flock communicate with the rest of the flock and navigate by keeping a close flocking distance. A pigeon's leadership is defined by the number of times a specific bird speaks with the other pigeons in a given random population.

The researchers in [11] examined the safety of medicinal images in the IoT environment through the advanced cryptographic method with metaheuristic optimizer-based approaches. For growing the security level of encrypting as well as decrypting procedures, the optimum key would be selected via hybrid swarm optimization, viz., GSO, and PSO in elliptic curve cryptography. In [12], the dual encryption process is exploited for encrypting medicinal images. At first, Blowfish Encryption is taken into account and signcryption procedure is employed to authorize the encryption method. Next, the Opposition-based Flower Pollination (OFP) is exploited to upgrade the public and private keys. Blowfish algorithm encrypts data and expands keys. P–array and S–boxes are used at the beginning of key expansion with numerous subkeys, requiring precomputation before encryption or decryption. P–array has 18 32-bit subkeys, P1 to P18. This section converts 448-bit keys into 4168-byte subkey arrays—The second stage of the Blowfish algorithm encrypts data, starting with a 64-bit plaintext block and ending with a 64-bit ciphertext. First, identical 64-bit segments 39. Second, the exclusive or–operation (XOR) between the initial 32-bit block termed left (*L*) and the first P-array. The 32-bit data from Step 2 is passed to the F function, which permutes it into a 32-bit block segment and XORs it with the right (*R*) 32-bit block of the main 64-bit plaintext. After XOR, *L* and *R* segments are exchanged for Blowfish iterations. P1 through P18 are utilized in reverse order for decryption. An opposition-based flower pollination algorithm, also known as an OBFPA, has been used to solve problems involving the optimization of functions and the construction of structures in engineering. The enhancement is accomplished through the utilization of two primary optimization methodologies. The local self-adaptive greedy approach promotes the population's ability to exploit its resources, while the global opposition-based learning of the elite population increases the population's genetic variation. The 18 benchmark functions and two structure engineering design issues are used to validate an elite opposition-based flower pollination method.

This study devises a Pigeon Inspired Optimization with Encryption-based Secure Medical Image Management (PIOE-SMIM) technique. The proposed PIOE-SMIM technique intends in the design of secret share creation (SSC) and the encryption process. At the initial stage, the medical images are converted into a collection of 12 shares using the SSC approach. In addition, an elliptic curve cryptography (ECC) scheme is employed for the encryption process. In order to produce optimum keys involved in the ECC model, the PIO algorithm is exploited with the aim of maximizing the peak signal-to-noise ratio (PSNR). Finally, on the receiver side, the decryption and share reconstruction processes are performed to construct the original images. For demonstrating an enhanced performance of the PIOE-SMIM model, a comprehensive simulation study is made. In summary, the study's contributions are given in the following.

- To introduce a new PIOE-SMIM model for a secure image management system in the healthcare sector
- To employ the ECC-based encryption technique to securely transmit the medical images
- To design a PIO algorithm to optimally generate the keys for enhanced secure medical image transmission.
- To validate the performance of the proposed model against a benchmark medical dataset and examine the results interms of different measures.

The rest of the study is planned as follows.  Section 3 presents the proposed PIOE-SMIM model and Section 4 elaborates the result analysis. Finally, the concluding remarks are drawn in Section 5.

## 2. Literature Survey

Sambit et al. [13] examined secure medicinal images with a pair of subkeys, first a pair of subkeys are provided through the tent and chaotic logistic maps. Adoptive GSO with PSNR and correlation coefficient fitness function has been presented for choosing the optimum public and secret keys amongst the arbitrary number.

Hasan et al. [14] introduced an effective, lightweight encryption model to design a secured image coding approach for the medical sector. The presented method applies two permutation approaches for securing medicinal images. The presented method is evaluated, analyzed, and compared with the encrypted one in security and implementation time. In [15], DNA-based cryptography, as well as dual hyperchaotic map methods, are suggested to offer higher-level security for the medicinal image. The digital medicinal image is larger in size and requires computation time. Song et al. [16] proposed a rapid and real-time cryptosystem by the means of chaotic encryption and parallel computing. The concept is based on a “permutation-substitution” structure of a chaotic encryption system. During the substitution phase, encryption over adjacent images through an expanded cipher-blockchain (CBC) model ensures desired diffusion in the entire image set. During the permutation phase, the batch image is classified into different groups. Then, multi-thread generates permutation coordinates simultaneously in every group to save time, from which more than one image is shuffled at one go.

The researchers in [17] employ the chaotic tent map and SCAN concept for medicinal image encryption. It comprises of pixel rearranging stage, diffusion operation, and bit plane decomposition. The amount of data in the images is divided into eight binary planes with differing proportions. Low and high four-bit planes undergo shuffling by diagonal and spiral SCAN patterns. In [18], an Improved Chaos Encryption (ICE) is employed for improving the secrecy using arbitrary nature. The overall energy is computed on the image and a comparison is made with adaptive thresholds for segmenting the Lorenz module employed in the chaotic technique for improving the security approach.

In [19], the authors presented a V-net convolution neural networks (CNNs) model relying on the 4D hyperchaotic method to encode medicinal images. At first, the plaintext medicinal image deal with 4D hyperchaotic sequence image, including pseudorandom sequence generation, image segmentation, and chaotic system processing. Next, the V-net CNN model is utilized for training chaotic sequences to remove the periodicity of chaotic sequences. At last, the chaotic sequence image undergoes diffusion for changing actual image pixels for realizing the encryption process. Masood et al. [20] a light-weighted cryptosystem based on Chen's chaotic, Henon chaotic map, and Brownian motion system is proposed for encrypting the healthcare images with higher security. The experiment result shows that the presented system is a lightweight technique that could accomplish the anticipated security level to encrypt secure image-based patient datasets.

Block-based image processing and hyper-image encryption techniques were used in this study to secure images. A transformation algorithm reorganized the original image into the transformed image and then encrypted the encrypted image using the Blowfish algorithm, or hyper-image encryption techniques. According to the results, the correlation between image elements has been much reduced. A lower correlation and higher entropy were also found when the number of blocks was increased by using smaller block sizes. There is not a key generator in this algorithm. In order to protect the image, a hyper-image encryption method was utilized. IJSER International Journal of Scientific and Engineering Research, Volume 7, Issue 1, January-2016 96 ISSN 2229–5518 is unable to process this volume's enormous amount of data. International Journal of Science and Engineering Research Multimedia data may not be acceptable for text-based algorithms. The degree of correlation between different aspects of an image was greatly reduced using this approach.

In Matlab 2013, Blowfish Algorithm is used to encrypt and decrypt images. In this study, a secret-key block cypher named 64 bits Blowfish was proposed for picture encryption and decryption, to improve both security and performance. This algorithm is used to generate keys with a range of sizes ranging from 32 bits to 448 bits. A Feistel network is used, which repeats a simple function sixteen times. There are no known vulnerabilities in the blowfish algorithm. It also outperforms the current industry standard in terms of speed and efficiency MATLAB is used to develop and implement the proposed algorithm. As a result, the blowfish algorithm becomes more robust when the number of rounds is increased. Blowfish is an effective standard encryption algorithm because it has no known security flaws.

## 3. The Proposed Model

In this article, a new PIOE-SMIM model has been developed for accomplishing a secure image transmission system. The proposed PIOE-SMIM technique mainly focuses on the design of SSC and the encryption process. At the initial stage, the medical images are converted into a collection of 12 shares using the SSC approach. In addition, the ECC scheme is employed for the encryption process. In order to optimize the key generation procedure in the ECC model, the PIO technique is exploited to maximize PSNR.

### 3.1. Share Creation Process

Generally, an image is comprised of overlapping shares, from which the human optical system finds private images without primitive technology. At last, the count of shares is widely distributed. The homogenous model contains sensor hubs that are similar because it is farther from dimensions namely battery power and the devices are extremely complex to construct. Hence, an image generates ”N” shares for the security system. E-Secret picture distribution is a fine talent and skill that concerns itself with the security of significant color photographs by dividing the secret into numerous copies. This is done in order to ensure the integrity of the image. The core idea behind the technique is to convert the color image into a number of different unreadable format type shares. This will ensure that the information contained inside the image will remain hidden until it is once again combined with some sort of mathematical computation. Security, reconstruction precision, computation complexity, and storage requirements are the four conditions that can be used to evaluate a Secret Sharing Scheme's performance.

Encryption is the process of transforming information into a code that is only known to a select few, hence concealing the information's actual meaning. The process of encrypting and decrypting information is referred to as cryptography, which is a scientific discipline. In computers, data that has not been encrypted is referred to as plaintext, and data that has been encrypted is referred to as ciphertext. Decryption is the process of converting data that has been encrypted back into its original form. In most cases, decryption is simply the process of encryption done backward. It takes the encrypted information and decodes it so that only a user with the appropriate authorization may decrypt the data. This is because decryption requires a secret key or password. Encryption is the process of transforming a message that can be read into one that cannot be read, to prevent third parties who are not authorized from accessing the information. The process of turning an encrypted message back to its original format (which can be read by humans) is referred to as decryption. The communication in its original form is referred to as the plaintext message. At the initial stage, the medical images are converted into a collection of 12 shares using the SSC approach. The pixel values of images were determined and the RGB values get described by matrix (*R*_*m*_, *G*_*m*_, *B*_*m*_). The size of the matrix is equal to input image size (*P* ^∗^ *Q*). It is described by the following equation:

(1) $$\begin{array}{c} \text{Pixel}=\sum R+G+B, \end{array}$$

whereas pixel denotes the overall *R*_*m*_, *G*_*m*_, and *B*_*m*_ pixel values. All the pixels present in the input images undergo *n* transformed way named shares. The RGB shares are depending upon the pixel value that exists in the RGB image. The shares for RGB are symbolized by *R*_*s*_, *G*_*s*_, and *B*_*s*_.

(2) $$\begin{array}{c} R_{s}=\int_{1}^{k}\frac{\text{lim}}{k⟶1\text{ton}}R_{ab},\\ G_{s}=\int_{1}^{k}\frac{\text{lim}}{k⟶1\text{ton}}G_{ab},\\ B_{s}=\int_{1}^{k}\frac{\text{lim}}{k⟶1\text{ton}}B_{ab}, \end{array}$$

whereas *a* and *b* shows the location of the matrices, (*R*_*s*_, *G*_*s*_ and *B*_*s*_) signifies the RGB share, (*R*_*ab*_, *G*_*ab*_ and *B*_*ab*_) represents the component of the image pixel. In previous to share formation, the elementary matrix is required to be derivative according to the number of shares to be produced that is fixed by the user. Here, the elementary matrix amount is 2 and the share amount is 4. Next, the elementary matrix is acquired by the abovementioned process and is characterized by *B*_*M*1 and_*B*_*M*2_ correspondingly. In previous to making share, the succeeding process is implemented on the matrices *XR*_1_ and *XR*_2_.

(3) $$\begin{array}{c} XR_{1}=128\;-\;B_{M1},\\ XR_{2}=B_{M2}. \end{array}$$

The red band share is generated by XOR operation among the basic matrix and key matrix as shown in the following.

(4) $$\begin{array}{c} Rs1\;=\;XR_{1}⊕K_{M},\\ Rs2\;=\;XR_{2}⊕XR_{1},\\ Rs3\;=\;XR_{2}⊕Rs_{1},\\ Rs4\;=\;Rs1\;⊕R. \end{array}$$

In the reconstruction process, multiple shares are incorporated to generate the original images that are shown below:

(5) $$\begin{array}{c} R\;=\;Rs1\;⊕Rs2\;⊕Rs3\;⊕Rs4\;⊕Rs4\;⊕K_{M},\\ G\;=\;Gs1\;⊕Gs2\;⊕Gs3\;⊕Gs4\;⊕Gs4\;⊕K_{M},\\ B\;=\;Bs1\;⊕Bs2\;⊕Bs3\;⊕Bs4\;⊕Bs4\;⊕K_{M}. \end{array}$$

When the share is recreated, the encryption and decryption method with the ECC approach is exploited on each color band of the recreated share. All the band images are separated into blocks previous to encryption and decryption procedures. The block is divided into the size of 4 ^∗^ 4. From the abovementioned process, the share is produced and an encryption procedure is employed.

### 3.2. ECC Based Encryption

**Figure 1:**
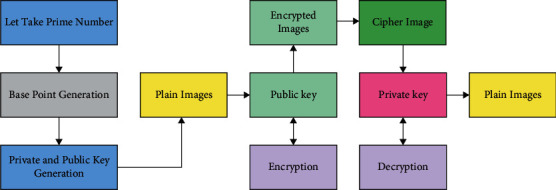
Process in ECC.

Once the shares are generated, the ECC scheme is employed for the encryption process. ECC is a public key cryptographic method relying on the algebraic architecture of elliptical curves through finite fields. It includes a small key through the non-EC cryptography for offering similar safety.  Figure 1 displays the working process of the ECC. At the ECC, the prime value is elected as *n*_*p*_ and the private key is preferred as *H* [21]. Next, it can be expressed in the following:

(6) $$\begin{array}{c} E=p{\left(i\right)}^{3}+u*p\left(i\right)+v, \end{array}$$

where *u* and *v* denote the constant value *u*=*v*=2. Once the state *X*=*Y* is satisfied, the optimum points are selected for the ECC. Next,  *X* and *Y* aredetermined as

(7) $$\begin{array}{c} X=\text{mod}\left(E,n_{p}\right),\\ Y=\text{mod}\left(p{\left(j\right)}^{2},n_{p}\right). \end{array}$$

Now, *p*(*i*,  *j*) denotes the point of the elliptic curve. *n*_*p*_ describes a prime number. The doubling technique is exploited for defining the *X* and *Y* values. The optimum point *P*_*e*_(*k*, *l*) and *P*_*f*_ indicates public key as given below:

(8) $$\begin{array}{c} P_{f}=H*P_{e}. \end{array}$$

In the encryption procedure, each share comprises a block and all the blocks are comprised of encrypted sections. The total number of blocks is indicated by *b*(*i*,  *j*) whereas *i* and *j*  indicates the row along with the column of the blocks. At this point, all the portions of information are delivered as an input to encrypt data. Data *D*_*x*_(*i*,  *j*) and *D*_*y*_(*i*+1, *j*) along with the point can be described as follows [22]:

(9) $$\begin{array}{c} C_{1}=H*P_{e},\\ C_{2}=\left(D_{x},D_{y}\right)+C_{1}. \end{array}$$

In decryption procedure, the private key (H) is employed for communication decryption whereas the point *C*_11_ is exploited to decrypt pixels.

(10) $$\begin{array}{c} C_{11}=H*C_{1},\\ C_{ij}=C_{2}-C_{11}. \end{array}$$

The *C*_*ij*_ shows the final result of the decryption procedure. From the outcomes of *C*_*ij*_, pixel values of IR and original color bands (RGB) are uniquely saved. Eventually, it can be expressed as follows:

(11) $$\begin{array}{c} F_{\text{image}}=R+G+B. \end{array}$$

Now, the keys exploited into encrypting and decrypting data are produced through the MRFO process that is deliberated in the succeeding subsection.

### 3.3. Optimal Key Generation

**Figure 2:**
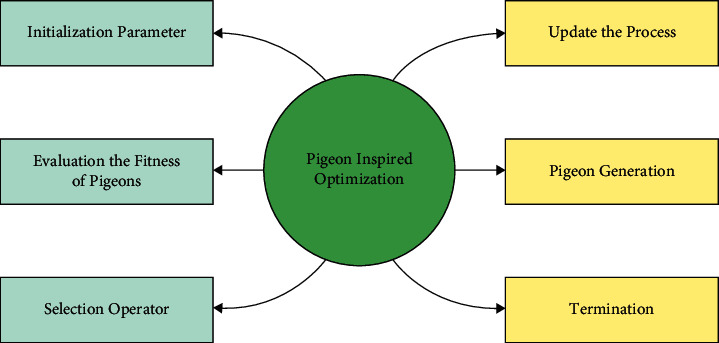
Flowchart of PIO technique.

To generate ECC keys optimally, the PIO algorithm is exploited to maximize PSNR. The POA has three operators: the compass, landmark, and map operators. During the map and compass operators, pigeons sense the geomagnetic field for the procedure the mapping to home [23]. In this recently developed technique, the map and compass operator model is presented based on the magnetic field and the sun, whereas the landmark operator model is presented based on the landmarks themselves. Map and compass operator: The birds would orient themselves by using the magnetic field of the Earth (also known as the CMT), which would also enable them to take over in the event that the environmental conditions no longer permitted optical orientation. This phenomenon is known as magnetoreception. Landmark operator: Pigeons rely on the neighboring landmarks in order to find their way as they go closer and closer to their destination. If they are familiar with the locations, they will bypass the intermediate stops and go straight to their destination. They will follow the pigeons that are familiar with the landmarks in the event that they are a considerable distance from the goal and do not know where the landmarks are located. Consider that searching space is *N* dimensional, and *i*^*th*^ pigeon of swarms is represented as *N* dimensional vectors *X*_*i*_ = (*X*_*i*,1_, *X*_*i*,2_,…, *X*_*i*,*N*_). The velocity of pigeons signifies the altered place of pigeons and is demonstrated as other *N* dimensional vectors *AC*. The previous visited place of *i*^*th*^ pigeons are signified as *P*_*i*_ = (*P*_*i*,1_, *P*_*i*,2_,…, *P*_*i*,*N*_). The global optimum place of swarms is presented as (*g*_1_,  *g*_2_,…,  *g*_*N*_). Every pigeon was flying dependent upon equations (12) and (13):

(12) $$\begin{array}{c} V_{i}\left(t+1\right)=V_{i}\left(t\right)\times e^{-Rt}+r\text{and }\times \left(X_{g}-X_{i}\left(t\right)\right), \end{array}$$

(13) $$\begin{array}{c} X_{i}\left(t+1\right)=X_{i}\left(t\right)+V_{i}\left(t+1\right), \end{array}$$

where *R* implies the map and compass factors, whereas *r* and refers to the arbitrary value in the range of [0, 1], *X*_*g*_ represents the global optimum solutions, *X*_*i*_(*t*) signifies the present place of pigeons at the time *t*, and *V*_*i*_(*t*) indicates the existing velocity of pigeon at iterations *t*.  During the landmark operator, each pigeon is ranked dependent on its fitness values. During every group, the amount of pigeons is upgraded by equation (14), whereas the number of pigeons were taken into account for evaluating the chosen place of centered pigeons, whereas each other pigeon changes its terminus as follows [24].

(14) $$\begin{array}{c} N_{p}\left(t+1\right)=\frac{N_{p}\left(t\right)}{2}, \end{array}$$

where *N*_*p*_ signifies the number of pigeons from the existing iteration *t*.  The place of the chosen terminus was measured by equation (1), whereas each other pigeon upgrades its place nearby equation (16).

(15) $$\begin{array}{c} X_{c}\left(t+1\right)=\frac{\sum_{X_{i}}\left(t+1\right)\times \text{Fitnes}\left(X_{i}\left(t+1\right)\right)}{N_{p}\sum \text{Fitness}\left(X_{i}\left(t+1\right)\right)}, \end{array}$$

(16) $$\begin{array}{c} X_{i}\left(t+1\right)=X_{i}\left(t\right)+r\text{and }\times \left(X_{c}\left(t+1\right)\times X_{i}\left(t\right)\right), \end{array}$$

where *X*_*c*_ signifies the place of centered pigeons.  Figure 2 demonstrates the flowchart of the PIO approach.

Optimization based on pigeons as seen in the images above, bio-inspired Swarm Intelligence Optimizer (SIO) is presented for the classification of satellite images [25–29]. Scientists have found that pigeons' orientation is primarily based on two operators that appear to utilizing particular rules. The pigeon's natural behavior, according to estimates, is that they travel long distances in search of food. They are equally adept at flying in dense woodland as they are at soaring through an open field. The pigeons' capacity to perceive the Earth's magnetic field, the sun's height, and visual cues like landmarks is another intriguing characteristic. Leading birds communicate and navigate with the remainder of the flock by flying side-by-side with each other. Researchers have shown that pigeons' amazing directing abilities are nearly entirely dependent on small magnetic particles in their beaks. Pigeons, in particular, have iron crystals in their beaks that give them a sense of direction. The trigeminal nerve appears to transmit messages from the nose to the brain via magnetite particles, according to research [30–33]. The pigeon's ability to detect the difference in height between the sun at the base and at the time of release has been regarded as evidence that the sun also plays a role in the pigeon's navigation. There is new evidence that pigeons can follow major highways, railroads, and waterways instead of flying straight to their destination, according to studies on the birds' behaviors. An optimum key selective method assumes that ‘fitness function' is the maximal key using PSNR for scrambling and unscrambling data in the medicinal images in IOT. The procedure was generated by the model of hybrid optimized for assessment. It can be demonstrated in the subsequent condition.

(17) $$\begin{array}{c} \text{fitness}=\text{maximum }\left\{\text{PSNR}\right\}. \end{array}$$

## 4. Experimental Validation

**Figure 3:**
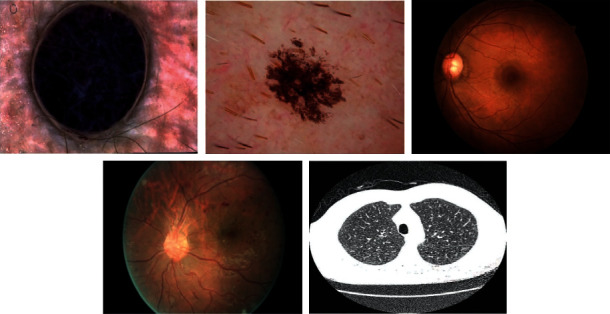
Sample medical images.

The experimental validation of the PIOE-SMIM model is tested using three medical datasets namely DR [25], dermoscopic [26], and lung [27] datasets.  Figure 3 demonstrates a sample test image.

To comprehend the share conception, a group of four shares (R, *G*, and B band) created to perform dermoscopic images is depicted in Table 1. The three rows from the table represent the shares of the RGB band correspondingly. As the shares outperformed in the table, it can be clear that the shares are not meaningful.

For understanding the share conception, a group of four shares (R, *G*, and B band) created to execute the DR image is exposed in Table 2. The three rows from the table signify the shares of the RGB band correspondingly. As demonstrated in the table, it can be clear that the shares do not convey any meaning.

**Figure 4:**
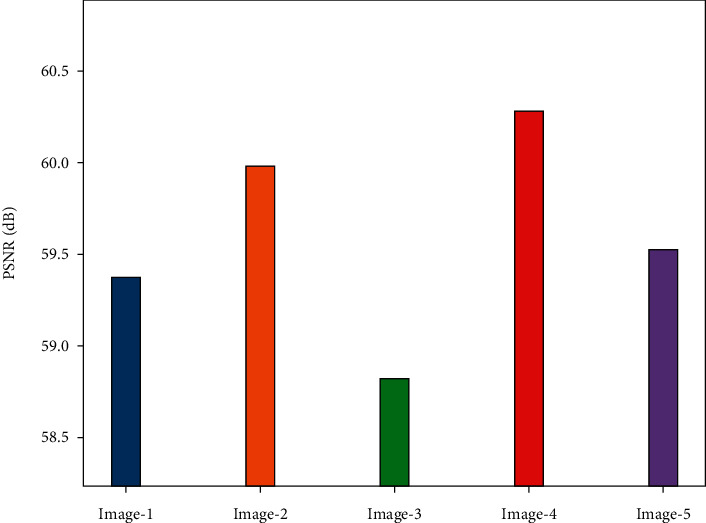
PSNR analysis of PIOE-SMIM technique with distinct images.

Table 3 provides a brief result examination of the PIOE-SMIM model under distinct test images.  Figure 4 highlights a brief PSNR investigation of the PIOE-SMIM model under a distinct number of test images. The figure indicated that the PIOE-SMIM model has resulted in increased values of PSNR. For instance, with image 1, the PIOE-SMIM model has provided a PSNR of 59.37 dB. Moreover, with image 2, the PIOE-SMIM model has provided a PSNR of 59.99 dB. Similarly, with image 3, the PIOE-SMIM model has offered a PSNR of 58.82 dB. Moreover, with image 4, the PIOE-SMIM model has resulted in a PSNR of 60.28 dB. At last, with image 5, the PIOE-SMIM model has accomplished a PSNR of 59.53 dB.

**Figure 5:**
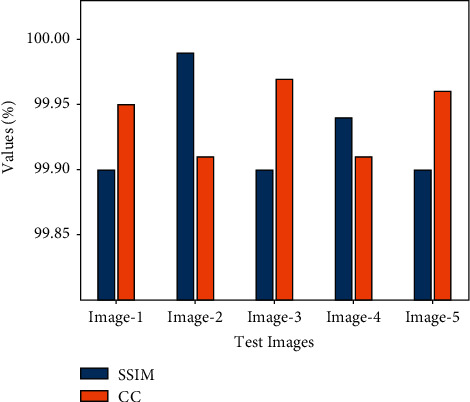
SSIM and CC analysis of PIOE-SMIM technique with distinct images.

Figure 5 demonstrates a detailed SSIM and CC study of the PIOE-SMIM model under a distinct number of test images. The figure designated that the PIOE-SMIM model has resulted in enlarged values of SSIM and CC. For instance, with image 1, the PIOE-SMIM model has offered SSIM and CC of 99.90 and 99.95. Also, with image 2, the PIOE-SMIM model has provided SSIM and CC of 99.99 and 99.91. Equally, with image 3, the PIOE-SMIM model has offered SSIM and CC of 99.90 and 99.97. Furthermore, with image 4, the PIOE-SMIM model has resulted in SSIM and CC of 99.94 and 99.91. Finally, with image 5, the PIOE-SMIM model has led to SSIM and CC of 99.90 and 99.96.

**Figure 6:**
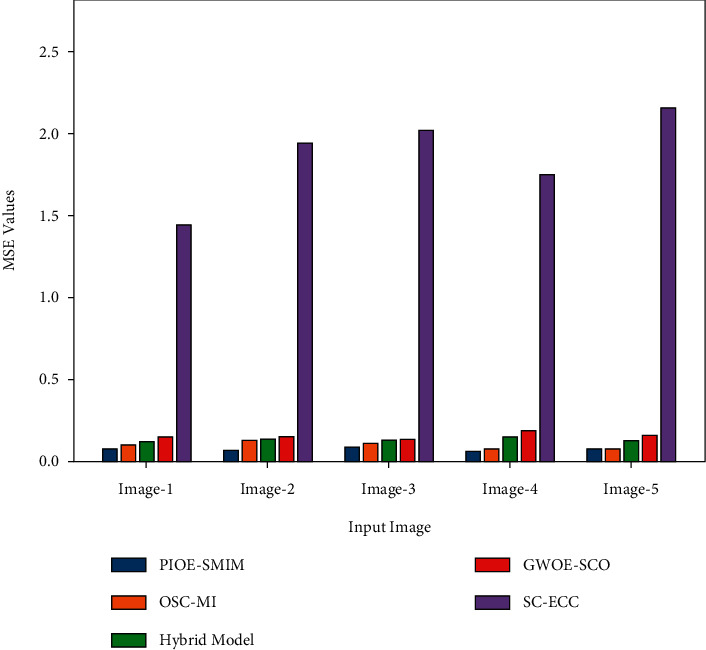
Comparative analysis of PIOE-SMIM technique with existing methods.

A detailed PSNR and MSE comparison study of the PIOE-SMIM model with other models is provided in Table 4 and Figure 6. The outcomes implied that the PIOE-SMIM model has accomplished enhanced values of PSNR and reduced values of MSE. For instance, with image 1, the PIOE-SMIM model has provided the least MSE of 0.0751 whereas the OSC-MI, hybrid, GWOE-SCO, and SC-ECC models have accomplished increased MSE of 0.1050, 0.1254, 0.1521, and 1.4460, respectively. At the same time, with image 5, the PIOE-SMIM model has offered a reduced MSE of 0.0725 whereas the OSC-MI, hybrid, GWOE-SCO, and SC-ECC models have gained improved MSE of 0.0812, 0.1280, 0.1542, and 2.1652, respectively.

**Figure 7:**
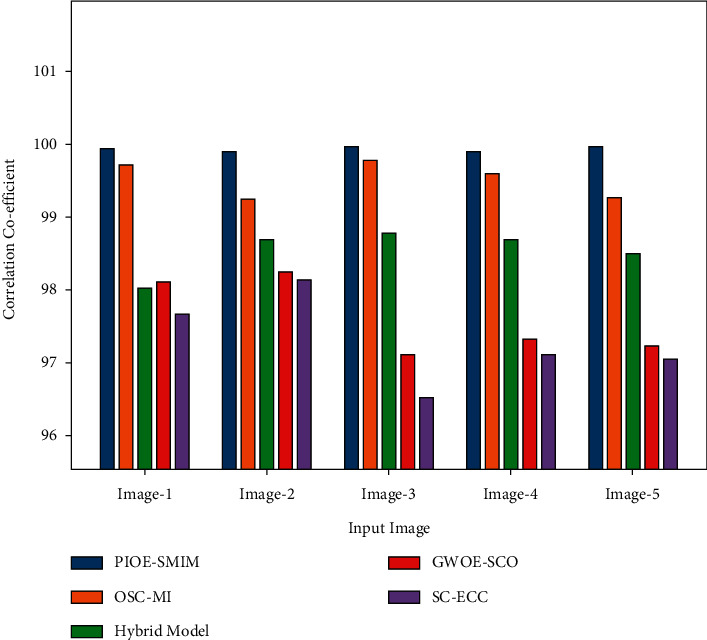
CC analysis of PIOE-SMIM technique with existing approaches.

A thorough CC comparison study of the PIOE-SMIM model with other models is provided in Table 5 and Figure 7. The outcomes portrayed that the PIOE-SMIM model has resulted in improved values of CC.

For instance, with image 1, the PIOE-SMIM model has provided a maximum CC of 99.95 whereas the OSC-MI, hybrid, GWOE-SCO, and SC-ECC models have accomplished minimum CC of 99.71, 98.03, 98.11,Similarly, with image and 97.68 respectively. In line with image 5, the PIOE-SMIM technique has offered superior CC of 99.96 whereas the OSC-MI, hybrid, GWOE-SCO, and SC-ECC models have gained decreased CC of 99.27, 98.51, 97.24, and 97.06, correspondingly [34].

**Figure 8:**
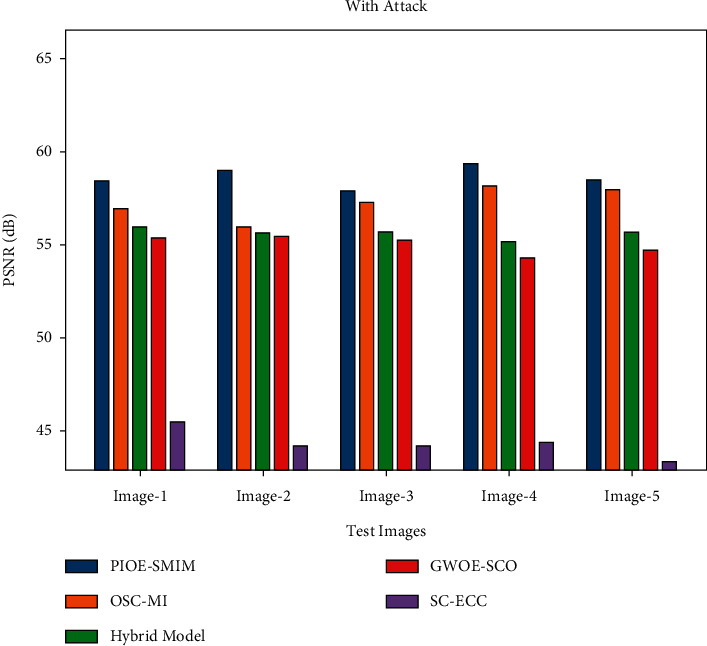
PSNR analysis of PIOE-SMIM technique with attacks.

Table 6 reports a detailed study of the PIOE-SMIM model with recent models under the existence and non-existence of attacks.  Figure 8 showcases the PSNR examination of the PIOE-SMIM model with other models under the existence of attacks. The results indicated that the PIOE-SMIM model has gained effectual outcomes with increased PSNR. For instance, with image 1, the PIOE-SMIM model has showcased an increased PSNR of 58.43 dB whereas the OSC-MI, hybrid, GWOE-SCO, and SC-ECC models have depicted reduced PSNR of 56.93 dB, 55.91 dB, 55.39 dB, and 45.53 dB respectively [35]. Similarly, with image 5, the PIOE-SMIM model has reported an improved PSNR of 58.52 dB whereas the OSC-MI, hybrid, GWOE-SCO, and SC-ECC models have exhibited lower PSNR of 57.96 dB, 55.68 dB, 54.76 dB, and 43.40 dB, respectively.

**Figure 9:**
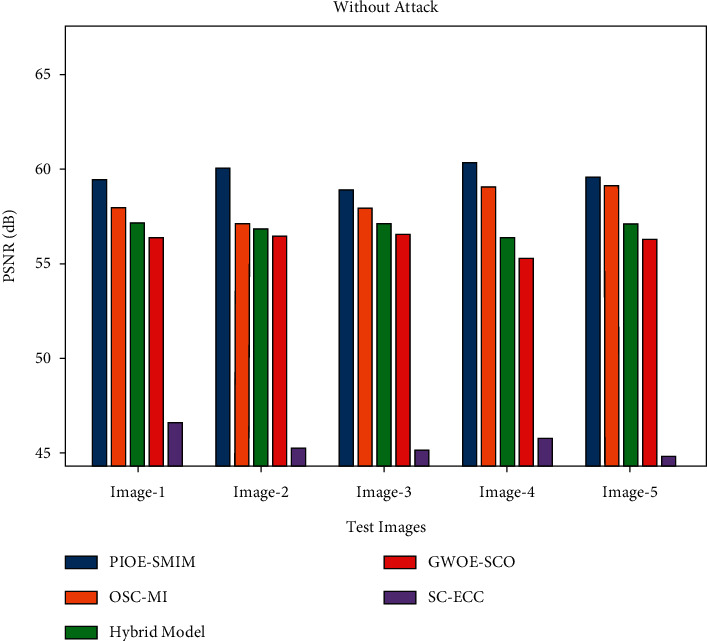
PSNR analysis of PIOE-SMIM technique without attacks.

Figure 9 illustrates the PSNR inspection of the PIOE-SMIM model with other models without attacks (WOA). The results designated that the PIOE-SMIM model has expanded capable outcomes with amplified PSNR. For instance, with image 1, the PIOE-SMIM model has showcased an improved PSNR of 59.37 dB whereas the OSC-MI, hybrid, GWOE-SCO, and SC-ECC models have portrayed compact PSNR of 57.92 dB, 57.15 dB, 56.31 dB, and 46.53 dB, respectively. Likewise, with image 5, the PIOE-SMIM model has stated enhanced PSNR of 59.53 dB whereas the OSC-MI, hybrid, GWOE-SCO, and SC-ECC models have displayed inferior PSNR of 59.04 dB, 57.06 dB, 56.25 dB, and 44.78 dB, respectively.

The abovementioned tables and figures implied that the proposed PIOE-SSIM model has accomplished enhanced security in medical image transmission. Therefore, the proposed PIOE-SSIM model can be employed in the real-time hospitals and healthcare institutions to securely transmit medical images.

## 5. Conclusion

In this article, a new PIOE-SMIM model has been developed for accomplishing a secure image transmission system. The proposed PIOE-SMIM technique mainly focuses on the design of SSC and the encryption process. At the initial stage, the medical images are converted into a collection of 12 shares using the SSC approach. In addition, the ECC scheme is employed for the encryption process. In order to optimize key generation process in the ECC model, the PIO algorithm is exploited to maximize PSNR. Finally, on the receiver side, the decryption and share reconstruction processes are performed to construct the original images. For demonstrating an enhanced performance of the PIOE-SMIM technique, a comprehensive simulation study is made and the results highlighted the supremacy of the PIOE-SMIM model over other techniques. In the future, blockchain technology can be applied to improve security in the healthcare sector. Blockchain is applied in healthcare and other industries. In healthcare, a Blockchain network stores and shares patient data among hospitals, labs, pharmacies, and clinicians. Blockchain can detect deadly medical blunders. This could improve medical data sharing's performance, security, and transparency. This tech analyses medical records. Blockchain can improve healthcare and other industries. Blockchain can uncover hazardous medical errors. Blockchain avoids dishonesty to enhance clinical trial results.

## Figures and Tables

**Table 1:** Visualization of multi-share creation scheme image 1.

Test image	Share 1	Share 2	Share 3	Share 4
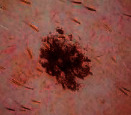	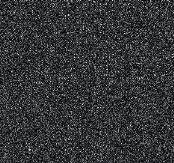	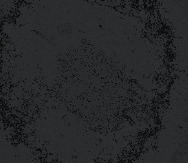	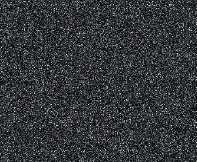	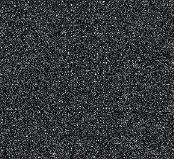
	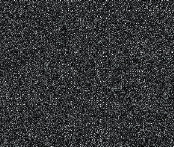	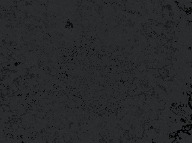	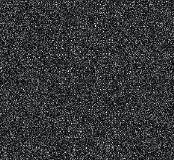	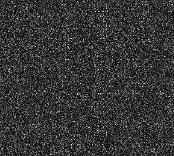
	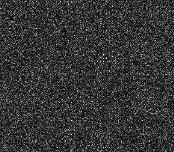	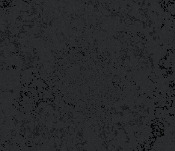	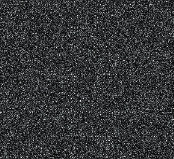	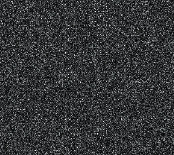

**Table 2:** Visualization of multiple share creation scheme image 2.

Test image	Share 1	Share 2	Share 3	Share 4
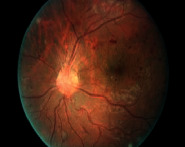	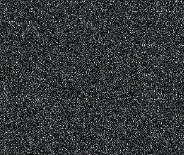	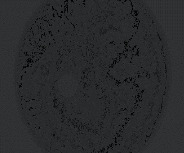	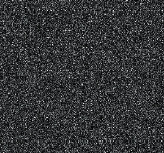	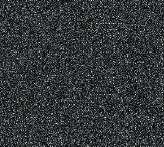
	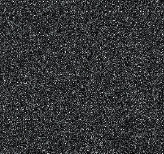	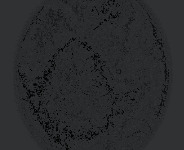	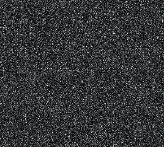	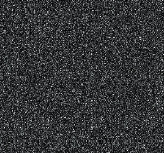
	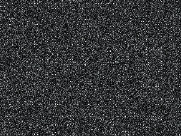	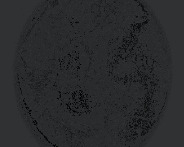	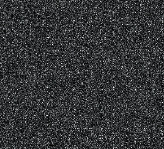	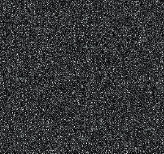

**Table 3:** Result analysis of PIOE-SMIM method with distinct measures and images.

Test images	MSE	RMSE	PSNR	SSIM	CC
Image-1	0.0751	0.2740	59.37	99.90	99.95
Image-2	0.0652	0.2553	59.99	99.99	99.91
Image-3	0.0853	0.2921	58.82	99.90	99.97
Image-4	0.0610	0.2470	60.28	99.94	99.91
Image-5	0.0725	0.2693	59.53	99.90	99.96

**Table 4:** Comparative analysis of PIOE-SMIM method with current methods in terms of MSE and PSNR.

Input image	PIOE-SMIM		OSC-MI		Hybrid model		GWOE-SCO		SC-ECC	
	MSE	PSNR	MSE	PSNR	MSE	PSNR	MSE	PSNR	MSE	PSNR
Image-1	0.0751	59.37	0.1050	57.92	0.1254	57.15	0.1521	56.31	1.4460	46.53
Image-2	0.0652	59.99	0.1287	57.04	0.1354	56.81	0.1479	56.43	1.9453	45.24
Image-3	0.0853	58.82	0.1054	57.90	0.1268	57.10	0.1395	56.69	2.0245	45.07
Image-4	0.0610	60.28	0.0812	59.04	0.1521	56.31	0.1952	55.23	1.7516	45.70
Image-5	0.0725	59.53	0.0812	59.04	0.1280	57.06	0.1542	56.25	2.1652	44.78

**Table 5:** Result analysis of PIOE-SMIM technique with recent algorithm in terms of CC.

Input image	PIOE-SMIM	OSC-MI	Hybrid model	GWOE-SCO	SC-ECC
Image-1	99.95	99.71	98.03	98.11	97.68
Image-2	99.91	99.24	98.71	98.25	98.14
Image-3	99.97	99.78	98.78	97.11	96.52
Image-4	99.91	99.61	98.71	97.32	97.13
Image-5	99.96	99.27	98.51	97.24	97.06

**Table 6:** Result analysis of PIOE-SMIM technique with recent approaches with respect to PSNR (with and without attack).

Test images	PIOE-SMIM		OSC-MI		Hybrid model		GWOE-SCO		SC-ECC	
	Attack	WOA	Attack	WOA	Attack	WOA	Attack	WOA	Attack	WOA
Image-1	58.43	59.37	56.93	57.92	55.91	57.15	55.39	56.31	45.53	46.53
Image-2	58.99	59.99	55.97	57.04	55.61	56.81	55.46	56.43	44.20	45.24
Image-3	57.86	58.82	57.27	57.90	55.71	57.10	55.28	56.69	44.18	45.07
Image-4	59.37	60.28	58.16	59.04	55.21	56.31	54.27	55.23	44.40	45.70
Image-5	58.52	59.53	57.96	59.04	55.68	57.06	54.76	56.25	43.40	44.78

## Data Availability

The manuscript contains all of the data.
